# Marine Fish Movement: home range sizes for commercially relevant species

**DOI:** 10.1038/s41597-024-03728-9

**Published:** 2024-08-10

**Authors:** Darcy Bradley, Alicia M. Caughman, Sandra A. Fogg, Reniel B. Cabral, Juan Mayorga, Whitney Goodell, Katherine D. Millage, Timothy D. White

**Affiliations:** 1 Bren School of Environmental Science and Management, University of California, Santa Barbara, CA USA; 2 Marine Science Institute, University of California, Santa Barbara, CA USA; 3 California Oceans Program, The Nature Conservancy, Santa Barbara, CA USA; 4 College of Science and Engineering, James Cook University, Townsville, QLD Australia; 5 Pristine Seas, National Geographic Society, Washington, DC USA; 6 Hawaii Institute of Marine Biology, Kaneohe, HI USA; 7 Global Fishing Watch, Washington, DC 20036 USA

**Keywords:** Behavioural ecology, Animal migration, Conservation biology

## Abstract

Estimates of home range sizes for marine fishes are essential for designing and assessing the effects of spatial wildlife conservation policies and management interventions. However, *in situ* studies of marine species movement are challenging and often expensive, resulting in a paucity of data on the home range size of the vast majority of marine fishes. Here, we develop a set of new datasets, which we have collectively named Marine Fish Movement, that synthesises published empirically evaluated home ranges reported for adult marine fishes that interact with fisheries and leverage these data to estimate home range sizes for unstudied species. The empirical data contain estimated home range sizes (km^2^) for 193 species across 63 family groups from 179 studies published between 1971 and 2022. We use a random forest regression model to estimate home range sizes (km^2^) for 664 fished marine species currently lacking home range estimates. Marine Fish Movement can inform spatial interventions including the design and management of marine protected areas and dynamic fisheries management to meet sustainability goals.

## Background & Summary

The study of animal movement in the ocean is constrained by logistical challenges associated with observing marine species *in situ*. Underwater direct observations by divers or snorkelers or tag-recapture via catch and release fishing long served as the primary windows into understanding space use by marine organisms. Recent advances in biologging technology have enabled the remote measurement of the movements and behaviour of free-ranging marine species, leading to a rapid expansion of animal movement ecology research in the ocean^1–3^. Satellite and acoustic telemetry now allow for the precise estimation of the space use of a broad range of taxa across coastal and open ocean ecosystems, and provide data that can be used to define home ranges among other spatial features of biological and ecological significance^1,4,5^.

A home range is the area an animal regularly uses during the course of its normal activities^6^. Estimates of animal home ranges are generally quantified through kernel density estimation^7,8^ to map utilisation distributions (UDs) across a landscape or geometric methods such as the minimum convex polygon (MCP)^9^. Resulting home range estimates are explicitly linked to location and habitat to describe an animal’s cognitive map of its “home”^10^, often delineated into individual kernels to distinguish core use from general or even total use areas (i.e., 50%, 95%, or 100% UDs or MCPs). Though size is just one of many components of a home range, it serves as a useful metric of animal movement for inclusion in spatial conservation planning and management. Species’ home range size is often a necessary parameter to design and evaluate marine protected areas^11–15^ and other spatial fisheries management interventions such as Territorial Use Rights in Fisheries (TURFs)^16^. For example, Green *et al*.^14^ recommend designing protected areas to be more than twice the size of the home range of focal coral reef fishes. Despite technological and statistical advancements in measuring and assessing home ranges, most marine species remain unstudied, including species targeted for fisheries and conservation management.

**Fig. 1:**
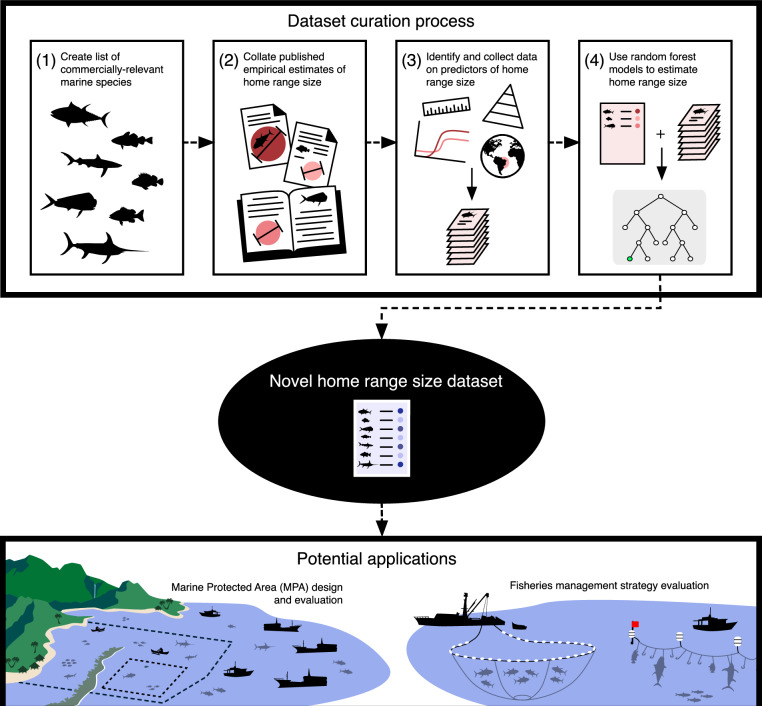
Conceptual schematic of the data curation process and the potential applications of the novel dataset. A targeted literature review of commercially relevant marine fishes was conducted to compile published home range size data; random forest regression models were used to estimate home range sizes for unstudied fishes. These data can be used to inform marine spatial planning efforts and management strategy evaluation for wildlife conservation and management.

Here, we provide a collection of novel datasets, Marine Fish Movement^17^, that contain home range size estimates for adult marine fishes that interact with fisheries as target or incidental catch. The primary home range size estimate dataset was developed using the following procedure: (1) create a species list of marine fishes that interact with commercial fisheries as target or incidental catch; (2) collate published empirical estimates of individual species’ home range sizes via targeted literature review; (3) identify known and hypothesised predictors of species home range size and collect and harmonise requisite data for each species; and (4) implement random forest regression models to estimate home range sizes for marine fishes lacking empirical evaluation (Fig. 1). Companion datasets including the empirical home range size estimates collected through the literature review and categorical movement indices are published as part of the Marine Fish Movement collection.

## Methods

### Data collection

#### Species list

Marine Fish Movement targets global marine fishes that are caught by commercial fisheries, as reported by Costello *et al*.^18^ (n = 744 species). These data include all fish stocks, which we have combined at the species level, from global fisheries (both within exclusive economic zones and the high seas; across all gear types; including industrial and small-scale commercial fisheries).

#### Empirical home range size estimates

Using the species list, we conducted a targeted search of peer-reviewed literature via Google Scholar for empirical studies and reviews reporting home range information (Keywords: “[species name]” AND “home range” OR “kern* utili* density” OR “KUD” OR “minimum convex polygon” OR “MCP” OR “geometric” OR “GEO” OR “move*”). Opportunistic searches were also conducted using reference lists from identified studies. Home range studies use a variety of observational and analytical approaches to estimate home range size. Field surveys conducted via satellite, radio, and acoustic telemetry, tag recapture, or direct observation for species with home ranges that were small enough that they could accurately be described *in situ* were retained. We included studies with home range estimates produced via kernel utilisation distributions (KUD), minimum convex polygons (MCP), and other geometric estimation techniques (GEO) that defined home range as 75–100% of an adult individual’s total utilised area. Study method was recorded, with studies using satellite, radio, acoustic telemetry, or mark-recapture data to estimate home range size preferred over studies using visual observations, except when the home range was small enough to be visually tracked. All home range size estimates were converted to km^2^.

**Fig. 2:**
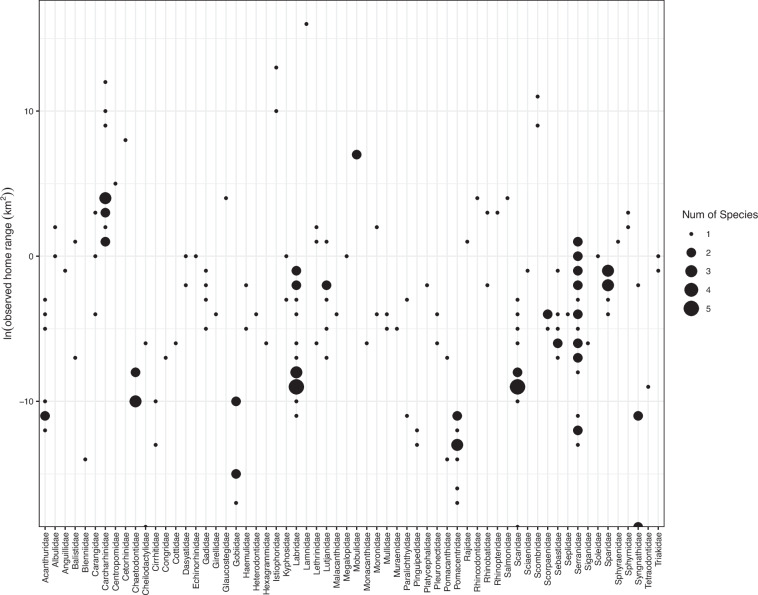
Overview of published home range sizes of marine fishes. Natural log of empirical home range sizes of commercially exploited fishes (n = 193) from 179 published studies shown by family group (n = 63).

For each study, we recorded the following information: species scientific name (Genus species), mean home range (km^2^), home range standard deviation (km^2^), home range percent (75–100%; i.e., the percent of the adult’s individual total utilised area), home range estimation method (KUD, MCP, GEO), observation method (direct observation, mark-recapture, telemetry), sample size (number of individuals), study duration (days), study location, reference. Note that standard deviations were inconsistently reported and home range estimates were often reported as averages. Consequently, we were often unable to independently calculate standard deviation from reported home range estimates. When mean home range was not reported, we derived it from the individual home range sizes reported. In cases where multiple studies were conducted on the same species, we calculated a weighted geometric mean across studies using reported sample sizes. If the sample size was not reported (n = 21), it was assumed to be 1 to down-weight the contribution of these studies. Unreported sample sizes tended to be from studies of highly territorial and/or site attached species (e.g., damselfishes, gobies) in which the number of individuals were reported as a density (individuals m^−2^) or not reported at all. This resulted in data records for estimated home range sizes of 193 species, representing 63 family groups from 179 unique references (Fig. 2).

### Statistical analysis

#### Model parameterization

To estimate home range size for fisheries-relevant species without empirical data, we built and evaluated random forest regression (RFR) models^19^. Our goal was not to mechanistically derive the key drivers of home range size, but rather we sought an approach that maximises overall model performance and predictive power. Given this, we used the RFR machine learning approach, which is an ensemble learning technique that applies different regression functions across the predictor space as opposed to a parametric or nonparametric function across the full suite of independent variables, as is the case in traditional regressions.

To predict home range size (km^2^), we assembled independent variables known (e.g., Krueck *et al*.^20^) or hypothesised to influence home range size: maximum length (cm), trophic level, intrinsic population growth rate, species-level carrying capacity, geographic range, and a descriptive movement keyword (final model variables are shown in Table 1). A standard maximum length measurement is not available on FishBase^21^, so total length (snout to tip of tail), fork length (snout to fork of tail), and standard length (snout to last vertebra) were all accepted length measurements. For species without reported trophic levels, we used a linear regression model to predict trophic level from reported food trophic level values. For species missing both trophic level and food trophic level, a genus level mean was used to impute missing data. Intrinsic growth rate (*r*) reported by species and carrying capacity (*K*) reported by stock were taken from Sala *et al*.^22^, with stock level *K* summed across stocks to get a species-level *K*. Geographic range size reported by predicted AquaMaps^23^ range maps based on a 0.5 threshold probability of occurrence was also included for each species. The descriptive movement parameter dataset (both a keyword and a categorical classifier were tested) was assembled from a systematic keyword search across three databases that were searched sequentially: FishBase^21^, the Food and Agricultural Organization (FAO) of the United Nations^24^, and the International Union for the Conservation of Nature (IUCN) Red List of Threatened Species^25^ (Table 2). After imputing missing trophic values, species missing any predictor variables were excluded from the model. The full set of estimation parameters were available for 664 of the 744 commercially caught fish species, including 70 of the 193 species with empirical home range size estimates, which we merged by species name in R (version 4.1.3) using the *tidyverse*^26^ package.

**Table 1:** Estimation parameters and data sources used in the random forest regression model.

Estimation parameter	Data source
Maximum length (cm)	FishBase^21^
Trophic level	FishBase^21^
Intrinsic growth, *r*	Sala *et al*.^22^
Carrying capacity, *K*	Sala *et al*.^22^
Geographic range	AquaMaps^23^
Movement keyword	This paper

**Table 2:** Categorical movement classification system developed for the random forest regression model.

Movement keyword	Movement classifier	Description	FishBase/FAO/IUCN keywords
sedentary	low	adults are sessile (e.g., toadfish), burrow/crawl/attached with limited movement; sensu Welch (18)	“sessile”; “burrow”; “limited movement”; “sedentary”; “home ranging”
territorial	low	adults are territorial with limited territory size	“territorial”; “home ranging”
habitat_reef	medium	adults are associated with reef habitat (coral reef, rocky reef); generally found in coastal waters	“coral”; “rock”; “reef”; “inshore reef”; “associated with reefs”; “reef-associated”
habitat_coastal	medium	adults are associated with non-reef coastal waters (lagoons, estuaries, rivermouths, seagrass beds)	“coastal”; “inshore”; “lagoon”; “brackish waters”; “seagrass beds”; “continental shelf”; “pelagic inshore”
habitat_benthic	medium	adults are associated with the benthos	“sandy bottom”; “benthic”; “mud”; “demersal”
habitat_deep	medium	adults are associated with deep ocean habitat (>100 m)	“deep water”; “outer continental shelves and upper slopes”; “bathydemersal”; “benthopelagic”
hms	high	adults are highly migratory species	“highly migratory species”
migratory	high	adults undergo regular migrations >50 km	“strongly migratory”; “extensive migrations”; “migrant”
pelagic	high	adults move throughout the pelagic zone	“pelagic”; “oceanic”; “open sea”; “offshore”; “free-living”
deep	high	adults are transient at depths >100 m	“bottom browser”

Detailed results and references are published as Data Record #3 along with this publication.

#### Model implementation, validation, and testing

Empirical home range data were split into a training (48 species) and a testing (22 species) dataset for model fitting and evaluation, stratified by family to get a representative sample of all scientific families present within the empirical data in the training dataset. We explored alternative stratification procedures, including stratifying by movement keyword (see Table 2), but this approach did not perform as well as the family level stratification and so was not retained for the final model. All numeric predictors were normalised, and home range size values were log-transformed to prevent an over-influence of large values on model predictions. Cross validation was used to determine the best values for the following hyperparameters: the number of trees in the model, the number of predictors in each sample, and the minimum number of data points in each node required for node split for each model tested. We examined root mean squared error (RMSE) and R^2^ values for predicted test data to assess model performance and for model selection (retaining the model with the lowest RMSE and highest R^2^; Table 3). Cross validation was also used to generate an estimate of out-of-bag error (OOB RMSE; error when predicting samples that were not used in training a specific tree). All models were implemented using the *tidymodels*^27^ and *ranger*^28^ packages, and variable importance was assessed using RMSE dropout loss implemented via the *DALEX*^29^ package in R.

**Table 3:** Model selection for the random forest regression testing model.

Model	Strata	Test RMSE	Test R^2^	Same ORD (%)	1 ORD difference (%)	>1 ORD difference (%)
*hr ~ r* + *K* + *m* + *l* + *t*	move	46363.82	0.43	17.39	8.70	73.91
*hr ~ r* + *K* + *m* + *l* + *t*	family	4989.64	0.77	4.55	18.18	77.27
log*(hr) ~ r* + *K* + *m* + *l* + *t*	move	4.82	0.24	21.74	39.13	39.13
log*(hr) ~ r* + *K* + *m* + *l* + *t*	family	3.80	0.26	**27.27**	**50.00**	**22.73**
**log*(hr) ~ r*** + ***K*** + ***m*** + ***l*** + ***t*** + ***f***	move	4.83	0.24	21.74	34.78	43.48
**log*(hr) ~ r*** + ***K*** + ***m*** + ***l*** + ***t*** + ***g*** + ***f***	move	4.61	0.29	13.04	43.48	42.48
log*(hr) ~ r* + *K* + *m* + *l* + *t*	move	4.60	0.29	113.04	43.48	42.48
**log*(hr) ~ r*** + ***K*** + ***m*** + ***l*** + ***t*** + ***g***	family	**3.32**	**0.44**	22.73	45.45	31.82

RFR model specification along with testing sample stratification procedure (taxonomic family, “strata family,” or movement classifier, “strata move”), test root mean squared error (RMSE), test R^2^, and percent of predictions that are the correct order of magnitude (ORD; i.e., same for observed and predicted), within 1 order of magnitude of the observed value, or >1 order of magnitude different from the observed value are shown. Bold has been used to indicate the best performing indicator across models; we selected the best estimation model based on the lowest RMSE and highest R^2^ of the models predicting the natural log of home range size. Parameter abbreviations: *hr* = home range; *r* = growth rate; *K* = carrying capacity; *m* = movement keyword; *l* = length; *t* = trophic level; *f* = family; *g* = geographic range.

**Fig. 3:**
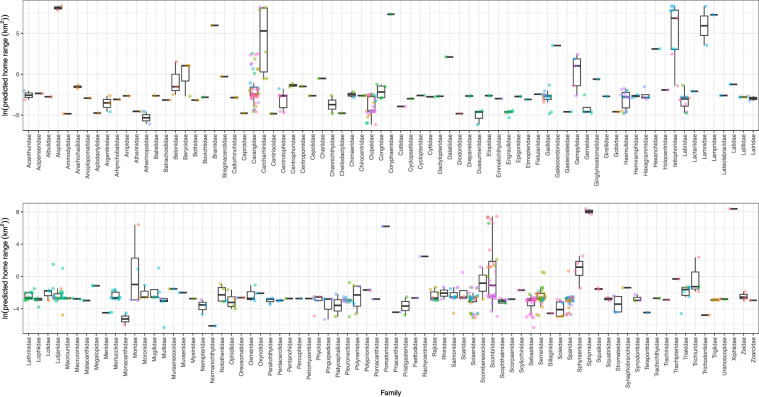
Estimated home range sizes of marine fishes. Natural log of home range sizes of commercially exploited marine fishes (n = 664) shown by family group (n = 151) and coloured by individual species estimated via a random forest machine learning model.

Model performance for training was deemed suitable with RMSE = 2.18 and R^2^ = 0.88. We used cross validation to find a mean R^2^ value across the training folds and then examined model accuracy within an order of magnitude by back-transforming predicted home range sizes and comparing them to empirical home range size estimates. These order of magnitudes are binned (i.e., 0.14 km^2^ = 10^−1^ = order of magnitude of −1, and 109 km^2^ = 10^2^ = order of magnitude of 2). The model was determined to be reliably generalizable with the mean R^2^ from cross validation equal to 0.51 (SE: 0.06) and an OOB RMSE of 4.39, only slightly higher than the full model RMSE (see below). We then used the same specification and procedure of the RFR model to generate predictions for all 664 parameterizable species in our list of commercially relevant marine fishes reported in the Marine Fish Movement dataset, along with predictions of the 5th, 25th, 75th, and 95th quantiles as measures of uncertainty. Estimated home range sizes are shown in Fig. 3 by family group, with home range size values back-transformed from the log scale for interpretability; quantile estimates are reported in the published dataset.

**Fig. 4:**
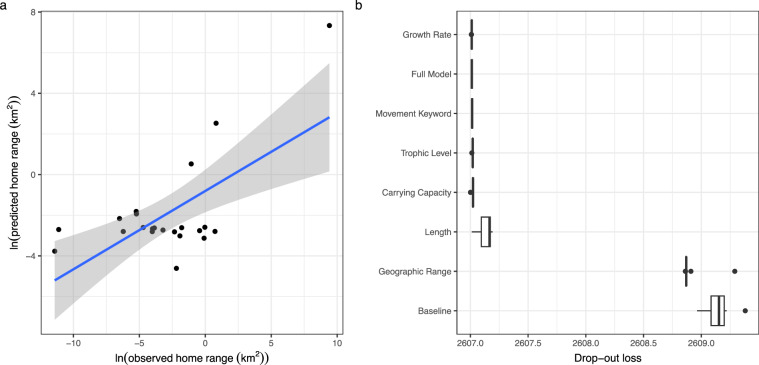
Model estimation and variable importance. Natural log of observed home range size estimates (from empirical data) compared to home range sizes estimated by the random forest regression (RFR) model (**a**). Variable importance via drop-out loss for each predictor variable in the (RFR) model (**b**); more important variables have a larger impact on model performance (i.e., larger drop-out loss) than less important variables.

Full model performance was again acceptable (RMSE = 3.32, R^2^ = 0.44). In general, the model underpredicted home range sizes (Fig. 4a). RMSE values are reported based on the log of the predicted data versus the log of the observed data. While more useful for summarising the entire model given our large range of values, this reporting method can impede interpretability. We therefore calculated RMSE for the back-transformed variables as well (RMSE = 2,281.9), which is inflated due to a single outlier that similarly obfuscates interpretability. Removing this outlier reduces the RMSE for the full dataset to 2.32. The influence of this outlier is further apparent when examining back-transformed RMSE values across home range size categories: high movers (home range size > 100 km^2^) = 10703; medium movers (1 km^2^ ≤ home range size < 100 km^2^) = 7.40; low movers (0.01 km^2^ ≤ home range size ≤ 1 km^2^) = 0.56; and lowest movers (home range size < 0.01 km^2^) = 0.1.

All variables were important for the model; intrinsic growth rate, trophic level, and carrying capacity were the most important model predictors, and length and geographic range were the least important (Fig. 4b). Across the testing data, 68.18% of predictions were within one order of magnitude of the observed home range size value (Table 3). Therefore, we recommend interpreting estimated home range sizes as being accurate within the predicted order of magnitude or using the quantile predictions, specifically the 25th and 75th quantiles, as a measure of uncertainty. These predictions improve markedly upon the previous classification of fish as either “low”, “medium”, or “high” movers.

Marine Fish Movement and associated code are publicly available (see Data Records section for access). Datasets and interactive data visualisations can be explored via an associated Shiny app (hosted at: https://emlab-ucsb.shinyapps.io/fish_homeranges/).

## Data Records

Marine Fish Movement contains three novel datasets and source code, which have been published as Marine Fish Movement to Dryad (data)^17^ and Zenodo (code)^30^. The repository contains the following files:

1. “homerange_rf_predictions.csv” contains the estimated home range sizes from the random forest regression model and associated data.
2. “empirical_homerange.csv” includes the results from the targeted literature review.
3. “movement_classification.csv” contains the detailed results from the movement classification system shown in Table 2.

A “README.md” file also published to Dryad provides additional descriptive information and metadata for each data file.

## Technical Validation

To validate published empirical home range size estimates, we had four independent reviewers (TW, SF, KDM, and DB) collect and cross check entries. Home range size averages across a reported sample and standard deviations were similarly calculated by independent reviewers. A fifth reviewer (AMC) did a final check of all recorded empirical home range sizes and associated data and calculations. Because charismatic species are often the subject of empirical home range studies, multiple independent studies were concentrated on a handful of species. For these species, a geometric mean home range size was calculated using study sample size (i.e., number of individuals per study) as the scaling factor.

Taxon names are inconsistently reported across studies and databases. To effectively merge species level information across our various datasets for model implementation, we used the World Register of Marine Species (WoRMS)^30^ as our reference taxonomic database and the following validation procedure:

- Get scientific names from WoRMS^31^, FishBase^21^, Encyclopedia of Life^32^ (EOL), Catalogue of Life (COL)^33^, and Global Biodiversity Information Facility^34^ (GBIF) using the *taxize*^35^ R package.
- Resolve scientific names starting with WoRMS, then FishBase, then EOL, then COL, then GBIF, if the name is missing from the prior sources.
- Cross check and filter resolved scientific names from *taxize* against the valid names in WoRMS using the *worms*^36^ R package to remove subspecies or synonyms. This resulted in resolved scientific names for 189 out of 193 validated species names.
- For the remaining four unresolved scientific names, filter out “ambiguous” records, for which a resolved scientific name has multiple records. This occurred for two species and the records with the accepted valid name from WoRMS was retained.
- The valid name of an additional two records were unaccepted due to an outdated species name. For these cases, the resolved scientific name was used, resulting in the validation of all 193 species.

Details and associated code are available in “clean_spp_names.Rmd” published as part of Marine Fish Movement^30^.

### Considerations for application

This paper presents Marine Fish Movement, a dataset containing predicted home range values for a wide range of marine fish species. We note two key considerations when using Marine Fish Movement: First, home range studies are expensive and dominated by large, charismatic taxa (i.e., sharks, tuna), as evidenced by the empirical data collected to develop our predictive model. As a result, home range model training and testing are based on a limited sample that does not represent all marine taxa. We therefore recommend data users interpret results at the order of magnitude level, where individual RMSE values were lowest and indicative of good model fit. Note that order of magnitude home range estimates are trained on the log scale due to a large range of values and skewed distribution; predictions at this scale have been back-transformed for interpretability.

Second, the degree of “accuracy” of the results depends to some extent on the type of movement characteristic of the species (e.g., sedentary, territorial, migratory, associated with reefs, pelagic – see Table 2 for details). For species with relatively small home range values, even seemingly small RMSE may be too large for some analyses, while for high mobile species, the same RMSE value may be acceptable. For very low movement fish (home range values < 0.01), data users are advised to explicitly consider their tolerance for error for a given application. For example, for small coastal MPAs, calculating minimum size to protect low movement species using home range values likely requires precision beyond that available for some estimates reported here. On the other hand, approaches using species area of occurrence and adult movement to determine appropriate sampling design for underwater monitoring may be more tolerant of error.

Taken together, practical application of Marine Fish Movement will, as with any data, require consideration of appropriate error and uncertainty. By publishing all model code^30^ and input data^17^, we hope that Marine Fish Movement can continue to be improved as additional marine fish home range estimates become available. At the same time, additional studies of key model parameters, including trophic level and intrinsic population growth rate, will further improve estimates of home range size for species lacking empirical assessment. The addition of new model parameters known to affect species movement extent, such as philopatry^37^, could further improve model performance. Notwithstanding these improvements, Marine Fish Movement can immediately be used to inform the design and management of a variety of ocean interventions, including large- and small-scale MPAs that seek to protect species within their borders, MPA networks that require an assessment of habitat connectivity from adult fish movement, TURF design to balance protection with production objectives, and dynamic spatial fisheries management to avoid endangered, threatened, and protected bycatch species and meet sustainability goals.

## Data Availability

All code associated with the Marine Fish Movement data^17^ are available as a zip file, “marine_fish_movement_code.zip” hosted by Zenodo^30^): 1. *data-prep* folder with two R Markdown scripts: (i) “clean_spp_names.Rmd” to clean up species taxonomy, and (ii) “randomforest_dataprocessing.Rmd” for harmonising and processing model variables. 2. *analysis* folder with a single R Markdown script, “homerange_randomforest.Rmd” that sets up and implements the random forest regression model. 3. *README.md* providing further relevant details. Code can be reproduced using R version 4.1.3.
